# Atezolizumab-induced vanishing bile duct syndrome: a case report

**DOI:** 10.3389/fonc.2025.1637847

**Published:** 2025-09-11

**Authors:** Carlos Tomás Noblejas Quiles, José Antonio Macías Cerrolaza, Javier David Benitez Fuentes, Laura López Gómez, Manuel Sánchez Cánovas, María Nevado Rodríguez, Miguel Martín Cascón, Isabel Vigueras Campuzano, Asunción Chaves Benito, Antonio David Lázaro Sánchez

**Affiliations:** ^1^ Department of Internal Medicine, Morales Meseguer General University Hospital, Murcia, Spain; ^2^ Department of Medical Oncology, Morales Meseguer General University Hospital, Murcia, Spain; ^3^ Department of Medical Oncology, Elche General University Hospital, Alicante, Spain; ^4^ Department of Intensive Care Unit, Morales Meseguer General University Hospital, Murcia, Spain; ^5^ Department of Pathological Anatomy, Morales Meseguer General University Hospital, Murcia, Spain

**Keywords:** Vanishing bile duct syndrome, Atezolizumab, immune-related adverse events, non-small cell lung cancer, case report

## Abstract

Vanishing bile duct syndrome (VBDS) is a rare but potentially fatal cause of intrahepatic cholestasis, usually associated with autoimmune, infectious or drug-induced etiologies. We present the first documented case of VBDS induced by Atezolizumab, an immune checkpoint inhibitor approved as adjuvant therapy in resected stage II-IIIA non-small cell lung cancer. A 63-year-old man developed cholestatic liver injury after three cycles of Atezolizumab, with progressive jaundice and elevated bilirubin despite immunosuppressive therapy. The diagnosis was confirmed by liver biopsy, which revealed intrahepatic bile duct leakage in more than 50% of the portal tracts. Despite initial stabilization, the patient’s bilirubin levels continued to rise and liver transplantation was contraindicated. He was discharged with immunosuppressive and supportive treatment, under close follow-up. This case highlights the need for greater clinical awareness of rare immunotherapy-associated immune-mediated hepatotoxicities, and underlines the importance of histological confirmation in severe or atypical presentations.

## Introduction

Lung cancer is the leading cause of cancer deaths worldwide, with an estimated incidence of 234,580 new cases and mortality up to 125,070 in the United States in 2024 (1, 2).

According to histological classification, non-small cell lung cancer (NSCLC) is the most common type (85%) (3). Surgery followed by chemotherapy has classically been the standard treatment for NSCLC, both in early and locally advanced stages (II-IIIA). However, based on the results of the phase III IMpower 010 study, adjuvant Atezolizumab has been approved for use for one year after chemotherapy in patients with resected stage II-IIIA lung cancer without EGFR or ALK alterations, and with PD-L1 expression ≥50%. This strategy offers an increase in disease-free survival and thus redefines the therapeutic paradigm in this subgroup of patients (4, 5).

Although immunotherapy has been a true revolution in cancer treatment, its growing and recent application in oncology has revealed adverse effects whose frequency and long-term impact have yet to be clearly determined. This represents a problem both at present and in the near future, as the emergence of immune-mediated toxicities could restrict the available therapeutic options and consequently jeopardize the continuity of cancer treatment.

In this context, immune checkpoint inhibitor-induced liver damage is the third most frequent immune-mediated adverse event (15%), after dermatological and gastrointestinal toxicity. Although its incidence with these drugs in monotherapy varies between 5-10%, cases of severe hepatotoxicity are less common, affecting less than 2% of the patients (6).

Clinically, liver damage usually presents as a hepatocellular pattern in 65-80%, with asymptomatic hepatitis being the most common form, followed by a cholestatic pattern in 10-25% of the cases (7). Within this latter pattern, an unusual but potentially serious entity stands out: vanishing bile duct syndrome, whose rarity and clinical relevance justify the publication of this case.

## Case report

This is a 63-year-old male with a history of pharmacologically controlled arterial hypertension and type 2 diabetes mellitus, with a significant smoking history corresponding to a 40 pack-year cumulative exposure. The patient was evaluated by Internal Medicine in April 2023 for constitutional syndrome accompanied by diarrhea with hematic debris.

The initial study was based on a thoraco-abdomino-pelvic CT scan, which showed a solitary 7mm pulmonary nodule in the right upper lobe (RUL), and a colonoscopy showing signs of mild aphthous ileitis, suggesting the possibility of Crohn’s disease.

In May 2024, lobectomy of the RUL was performed with mediastinal lymph node sampling by videothoracoscopy (VATS), with a definitive diagnosis of pulmonary adenocarcinoma (ADC) pT1bN2M0, PD-L1 49%, triple negative, for which he was referred to Medical Oncology in June 2024. Simultaneously, he was studied by the Digestive Department and the diagnosis of Crohn’s disease could not be confirmed.

In July 2024, adjuvant chemotherapy was started with Cisplatin 80 mg/m^2^ and Pemetrexed 500 mg/m^2^ every three weeks for four cycles, during which time the diarrhea ceased and there was a tendency towards constipation. In October 2024, he was started on Atezolizumab 1875 mg subcutaneous injection every three weeks. However, after the third cycle the hematochezia reappeared, leading to a new re- evaluation colonoscopy in December 2024 and discontinuation of treatment.

Colonoscopy findings were compatible with distal ulcerative colitis, ruling out cytomegalovirus infection. Mesalazine, both oral and topical, was prescribed and, given the cases of Atezolizumab-induced ulcerative colitis reported in the literature (8), immune-mediated toxicity was suspected.

However, the clinical situation worsened, with an increase in the number of bowel movements, abdominal pain and the appearance of febrile peaks, leading to several visits to the emergency department. At the end of January, methylprednisolone 150 mg/day oral pulses were prescribed on an outpatient basis for three days, during which time the patient remained afebrile. In addition, Metronidazole 500mg/12h was added for seven days with transient improvement of the gastrointestinal symptoms.

**Figure 1:**
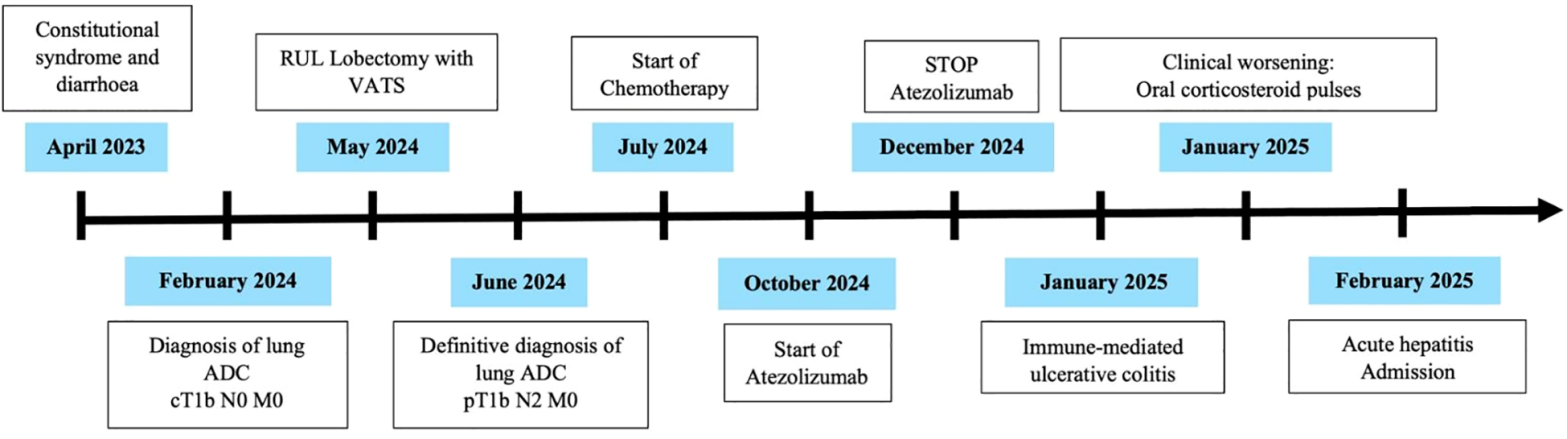
Timeline of clinical events.

Finally, at the beginning of February, he consulted the emergency department again due to recurrence of fever and stools with pathological products. Blood tests showed only an elevation of liver transaminases 10 times the upper limit of normal. Suspicion of acute hepatitis led to admission (**Figure 1**).

During his hospital stay, in addition to persistent fever, the patient developed mucocutaneous jaundice, choluria and acholia, with rapidly progressive cholestasis and mixed hyperbilirubinemia at the expense of direct bilirubin.

Firstly, after two pairs of blood cultures, empirical antibiotherapy was administered with Piperacillin-tazobactam 4/0.5g/6h and all possible hepatotoxic drugs were suspended. A complete blood test was performed with autoimmunity, antinuclear antibodies (ANA), anti-neutrophil cytoplasmic antibodies (ANCA), liver and kidney microsomal type 1 antibodies (LKM-1), anti-mitochondrial antibodies (AMA) and anti- smooth muscle antibodies (SMA), as well as immunoglobulin levels. These tests proved to be normal and viral serology was carried out, resulting negative.

After the initial complementary tests, an abdomino-pelvic ultrasound and a magnetic resonance cholangiopancreatography, no evidence of choledocholithiasis or intra- or extrahepatic bile duct dilation was found. However, the distal portion of the intrapancreatic common bile duct could not be visualized in detail and minimal ectasia of the Wirsung immediately proximal to the papilla was observed, so the study needed to be completed with an echo-endoscopy. The latter revealed a lymphadenopathy located in the pancreatic head, for which fine-needle aspiration was performed and the cytological evaluation resulted negative for malignancy.

**Figure 2:**
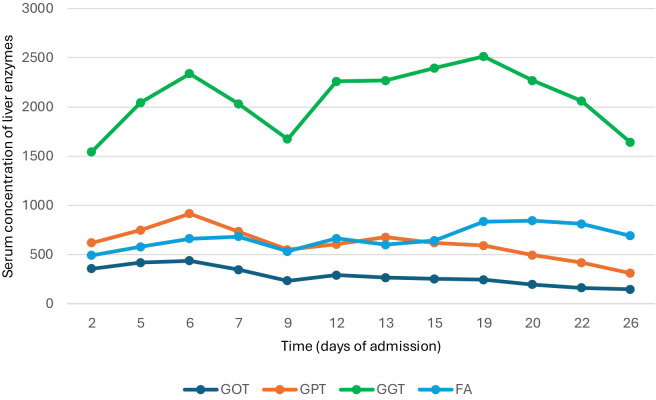
Variation in plasma liver enzyme concentration during hospitalization.

Nevertheless, given a liver profile that only worsened at the expense of a cholestatic pattern (**Figure 2**) with no dilatation of the biliary tree, along with the high suspicion of probable immune-mediated hepatitis, a liver biopsy was requested.

**Figure 3:**
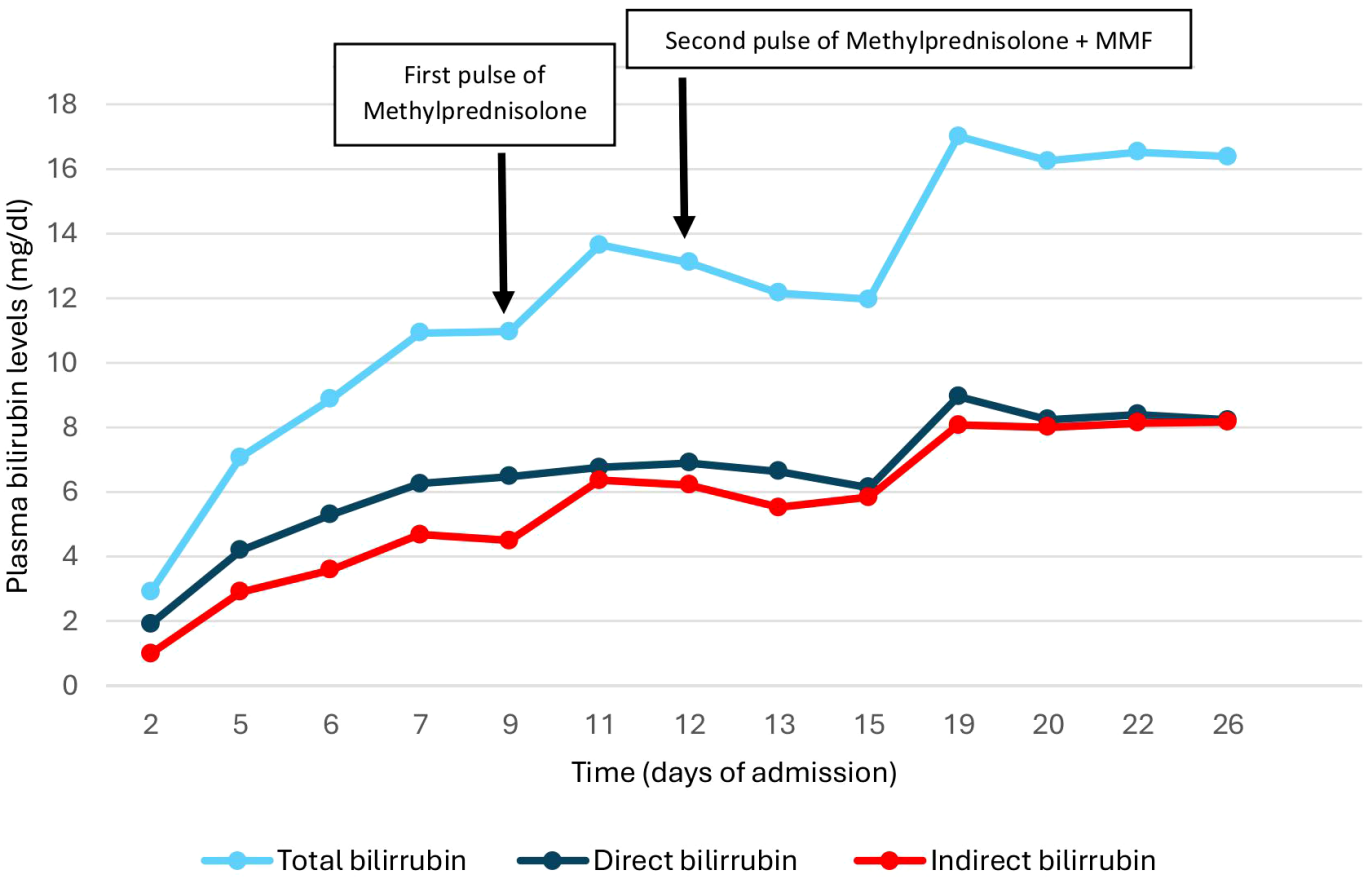
Changes in bilirubin levels during admission.

Afterwards, intravenous methylprednisolone 250 mg pulses were prescribed for 3 days and, after the first round, there was a decrease in bilirubin levels, which remained at a plateau (**Figure 3**). Considering this possible initial response, the administration of 250 mg intravenous methylprednisolone was repeated for another three days followed by Prednisone 30 mg/day. Immunosuppressive treatment was also administered with oral mycophenolate mofetil in increasing doses until reaching 1,000 mg every 12 hours. Clinically, the patient presented only mild pruritus, with no associated encephalopathy or coagulopathy.

**Figure 4:**
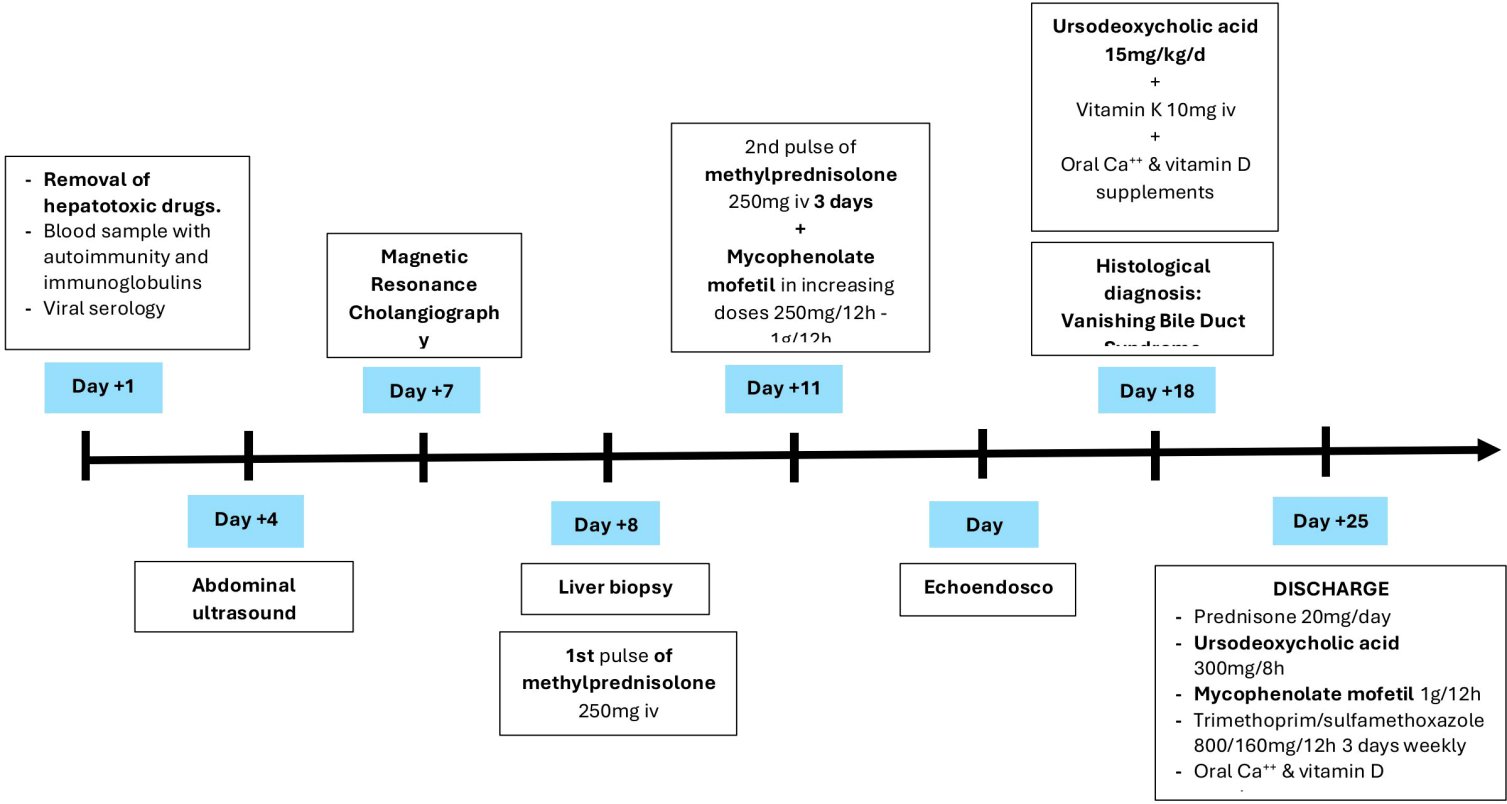
Timeline of events during hospital day.

The anatomopathological results of the liver biopsy confirmed the diagnosis of vanishing bile duct syndrome (**Figure 4**) induced by Atezolizumab, so ursodeoxycholic acid was started at a dose of 15mg/kg/12h with disappearance of pruritus, as well as intravenous vitamin K 10mg daily and oral calcium and vitamin D supplements.

Despite the initial improvement, bilirubin levels continued to rise rapidly and the case was discussed with the Liver Transplant Committee who, given the high risk of recurrence (70% at 5 years) and the need for a 5-year disease-free period (9), considered that the patient was not a candidate and therefore contraindicated this therapeutic alternative.

**Figure 5:**
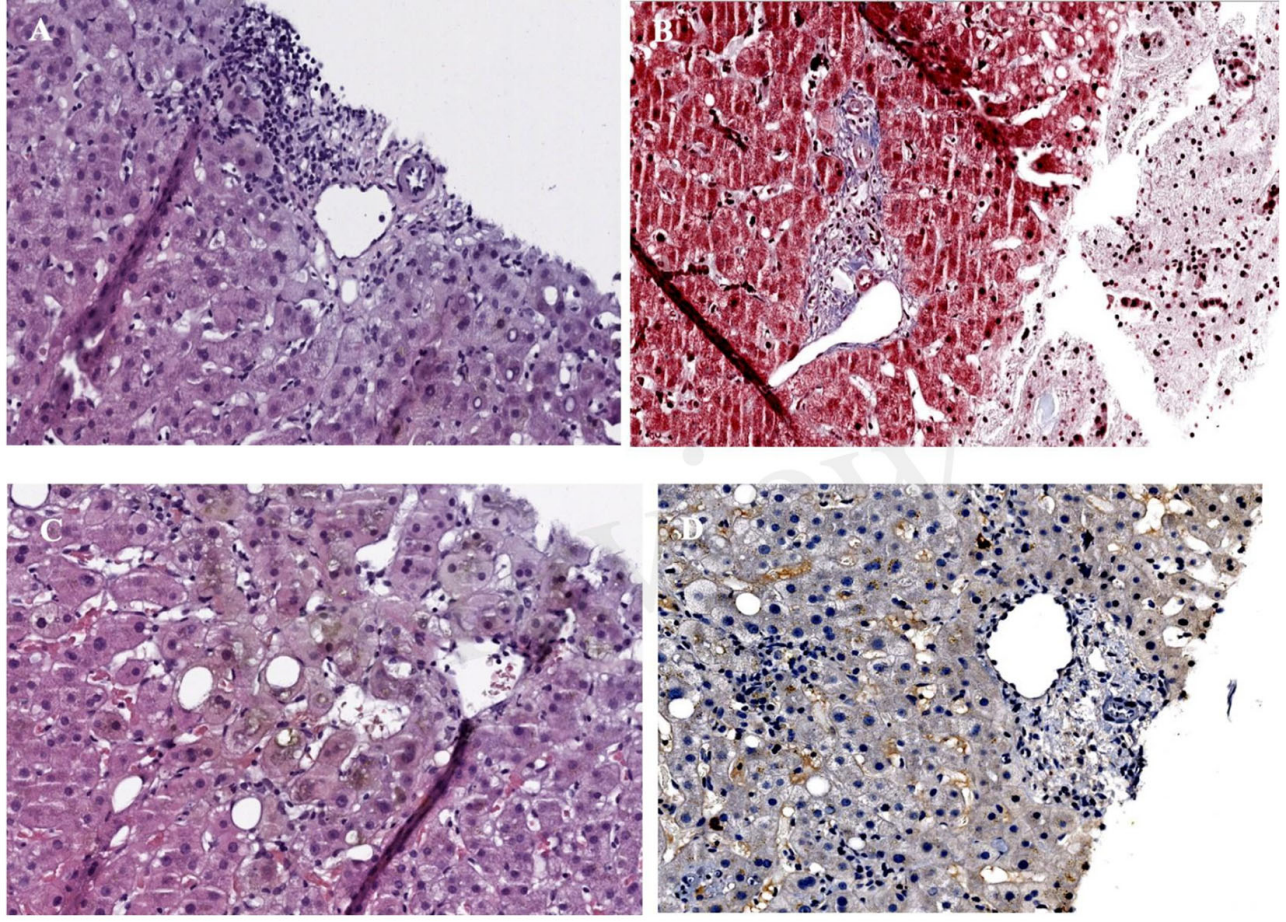
**(A)** Haematoxylin-eosin x40 (H-E): portal space with no bile ducts, only the branch of the hepatic artery and the branch of the portal vein are visible. **(B)** Masson x40: portal space with portal space with portal vein branch, several branches of the hepatic artery, as well as minimal fibrosis and inflammation, without distortion of the architecture. **(C)** H-E x40 centrolobulillar vein and bile pgment-laden hepatocytes reflecting intrahepatic cholestasis. **(D)** Immunohistochemistry with cytokeratin-19: absence of bile ducts in the portal space.

Finally, given his clinical stability, although with a guarded prognosis, the patient was discharged from the hospital with oral Prednisone 20 mg/24h, Mycophenolate Mofetil 1.000mg/12h, ursodeoxycholic acid 300mg/8h, prophylaxis with Trimethoprim/sulfamethoxazole 160/800mg/12h three times a week and oral calcium and vitamin D supplements, in addition to close follow-up in the Oncology Outpatient Clinic and support from the Autoimmune Unit of Internal Medicine (**Figure 5**).

## Discussion

This case highlights the relevance of recognizing that, although immunotherapy has redefined the therapeutic approach in lung cancer, it is important to consider the occurrence of infrequent adverse effects, but with a potentially lethal clinical impact, such as vanishing bile duct syndrome.

The initial approach in the differential diagnosis of cholestatic jaundice is based on establishing whether its origin lies in an intrahepatic or extrahepatic process.

Within the extrahepatic etiology, the presence of Charcot’s triad (fever, mucocutaneous jaundice and abdominal pain), described in up to 50-75% of cases, means that acute cholangitis must be ruled out first (10).

For intrahepatic jaundice, pharmacological hepatotoxicity must be excluded. In this particular case, the patient has been receiving Paracetamol, administered at a dose of 1g every 8 hours. While it is true that this drug is a well-established cause of liver damage and, in fact, is the main cause of acute liver failure, liver damage is dose-dependent and typically manifests as centrolobular necrosis which, at the analytical level, is reflected by a predominance of cytolysis. However, the dose received by the patient (3 g/day) is considered safe according to the Food and Drug Administration in the absence of underlying liver disease or chronic alcohol consumption (11).

On the other hand, although less frequent, both Metronidazole and Mesalazine have been implicated in isolated cases of liver damage, manifesting mixed or purely cholestatic hepatitis. Although the level of evidence supporting this probable association is weak, the possibility of liver injury induced by these drugs should be considered in the differential diagnosis (12, 13). In this particular case, there was no previous consumption of herbal products.

In the context of intrahepatic cholestatic jaundice, viral hepatitis should also be considered, with special emphasis on hepatitis A virus (14) and hepatitis E virus (15). Both can progress, in 5% and up to 60% respectively, to cholestatic hepatitis, characterized by persistent jaundice for more than three months. Despite its prolonged course, this form of presentation usually resolves spontaneously without significant sequelae. In this particular case, serology for hepatotropic viruses was negative.

Given the close relationship between inflammatory bowel disease, specifically Ulcerative Colitis present in almost 90% of cases, and Primary Sclerosing Cholangitis (16), it is imperative to include this entity in the differential diagnosis. In most cases (70%) there is an intrahepatic and extrahepatic involvement, being much more unusual, in less than 25% of cases, exclusively intrahepatic involvement (17). In this patient, imaging tests, especially magnetic resonance cholangiopancreatography, ruled out intra- and extrahepatic biliary tree dilatation, making it possible to rule out not only this disease, in addition to an incompatible liver biopsy, but also the other causes of extrahepatic cholestasis, including acute cholangitis.

Among the chronic autoimmune cholestatic diseases, primary biliary cholangitis, which affects only the intrahepatic bile duct and almost exclusively women, should be considered. However, the absence of antimitochondrial antibodies, detectable in more than 95% of cases, together with an atypical biliary histological lesion for this entity (18), allowed it to be excluded with a high degree of certainty.

Once all other entities have been excluded, immuno-mediated hepatitis should be considered, usually presenting 6–14 weeks after the start of immunotherapy with resolution within 4–6 weeks with appropriate treatment. Although the most common clinical picture is hepatocellular damage, usually early between the first and third immunotherapy cycle, in a quarter of cases it may present late, between the third and tenth treatment cycle, as a cholestatic pattern (7).

Staging of severity has direct implications for the management of immune- mediated hepatitis. For this purpose, the Common Terminology Criteria for Adverse Events (CTCAE) (6), traditionally used and accepted in oncology, classifies immune- mediated hepatitis into four grades according to the level of increase of transaminases and total bilirubin. In this case, an elevated total bilirubin greater than ten times the upper limit of normal was categorized as grade 4. In this subgroup, therapeutic management is based on permanent withdrawal of immunotherapy and initiation of intravenous corticosteroids at doses ranging from 1 to 2 mg/kg/day, which are considered the cornerstone of treatment (19).

In addition, routine blood tests with liver biochemistry should be performed every 1–3 days. In case of refractoriness to systemic steroids, defined by the absence of clinical and biological response within 3–7 days after starting corticosteroid treatment, mycophenolate mofetil at a dose of 1g/12h should be used first as second-line therapy, since it is the most studied drug and, therefore, the one for which most evidence is available, with response rates >80%. Another pharmacological alternative, although with little evidence, is Tacrolimus. As a last therapeutic option, in those who are refractory even to immunosuppressive treatment and/or rapidly progressive, isolated case series have used antithymocyte globulin and 5 sessions of plasmapheresis interspersed every 48 hours. Nonetheless, the lack of solid data on its efficacy precludes its widespread use (7).

Although liver biopsy is not recommended as standard, it can be crucial in the differential diagnosis of severe hepatitis, as in the present case. In this context, histological analysis of liver tissue confirmed the definitive diagnosis of vanishing bile duct syndrome.

VBDS is a rare, acquired but potentially severe form of chronic cholestatic liver disease (20). Although the pathogenesis remains unknown, it has been proposed to be an immune-mediated injury of biliary epithelial cells mediated directly by T lymphocytes (21), leading to apoptosis and thus progressive destruction of intrahepatic bile ducts, ultimately resulting in intrahepatic cholestasis.

As for the origin of ductopenia, although its association has been described with immune disorders (e.g. primary biliary cholangitis, primary sclerosing cholangitis, sarcoidosis and graft-versus-host disease), with infectious processes (e.g. cytomegalovirus, Epstein-Barr virus and hepatitis B and C virus), as well as with lymphoproliferative neoplasms (e.g. Hodgkin’s lymphoma) (22), it has classically been related to pharmacological etiology.

In fact, the first case, described in 1996, was secondary to the administration of Erythromycin (23), although others have also been reported such as Amoxicillin- Clavulanic acid, Trimethoprim-Sulfamethoxazole and Chlorpromazine (24). The medical literature has documented cases of this syndrome associated with the use of immunotherapy, particularly Pembrolizumab (25–28) in the context of metastatic NSCLC (25, 26), melanoma (27, 28) and mesothelioma (28). In most cases, liver toxicity manifested after the first treatment cycle, with the exception of one case in which the onset of liver toxicity occurred after the twelfth session (27). However, to date, no cases of VBDS directly induced by Atezolizumab have been reported, being this the first case.

The diagnosis is of exclusion and is established by histological examination. Therefore, in addition to clinical, analytical and serological evaluation to identify possible underlying causes and rule out extrahepatic bile duct obstruction, diagnostic confirmation requires histological identification of a loss of bile ducts in more than 50% of the portal spaces in a specimen with at least 10 portal tracts, obtained at least one month after the onset of liver damage (29). Another criterion in favor of this diagnosis is the temporal concordance between drug exposure and the onset of liver damage, usually between 1 and 6 months after the start of treatment.

From a biochemical point of view, the persistence of elevated alkaline phosphatase (>3 times the upper limit of normal) and hyperbilirubinemia for more than 6 months after drug exposure reinforces the diagnostic suspicion (24). Although moderate increases in transaminases may be observed, it is unusual for these to exceed 10 times the high threshold of normal.

Initial treatment, similar to that described in the literature for cases induced with Pembrolizumab, is based on ursodeoxycholic acid at a dose of 13–15 mg/kg per day for its cytoprotective, anti-apoptotic and immunomodulatory effects associated with methylprednisolone at 1 mg/kg or, less clearly, 2 mg/kg intravenously (28), although with limited scientific evidence on the impact on the natural course of the disease.

The prognosis is variable, although generally unfavorable, with the main determinant being the extent of ductopenia. In the available literature of cases with Pembrolizumab, four died, three of them despite an initial improvement of the liver profile; two from progression of the underlying oncological disease (28), one from non- neutropenic sepsis (25) and the remaining from acute liver failure (27). Only one patient survived with normalized liver biochemistry (26). Therefore, the natural history is unpredictable with two possible scenarios: gradual intrinsic recovery of the biliary epithelium may occur over months and/or years with progressive resolution of symptoms, or irreversible and progressive loss of bile ducts may occur, ultimately leading to cirrhosis (30).

In this case, both clinical and hepatic biochemical evolution is favorable, with normalization of serum bilirubin levels after 6 months of treatment with mycophenolate mofetil and ursodeoxycholic acid in combination with decreasing doses of corticosteroids, being currently 5 mg of oral prednisone in maintenance (**Table 1**).

**Table 1:** Comparison between Pembrolizumab and Atezolizumab-induced vanishing bile duct syndrome cases.

Study	Time to hepatotoxicity	Biopsy findings	Management	Outcomes
Gemelli et al.	11 days after the first administration of Pembrolizumab	Extensive intracanalicular and cellular cholestasis + severe ductal loss + mild lymphocytic inflammation	• Discontinuation of Pembrolizumab • High-dose steroid therapy (methylprednisolone 2 mg/kg IV) + MMF 1g/day + UDCA 600mg/day	Death from non- neutropenic sepsis
Masseti et al.	20 days after the first infusion of Pembrolizumab	Absence of interlobular bile ducts in >50% of portal tracts examined + prominent canalicular cholestasis + mild portal inflammatory infiltrate + no features of destructive cholangitis	Discontinuation of Pembrolizumab (no corticosteroids or other immunosuppressive therapies)	Progressive improvement of clinical and biochemical parameters over 16 weeks
Thorsteindottir et al.	After the twelfth infusion of pembrolizumab	Absence of bile ducts in the majority of the portal tracts + extensive intracellular and intracanalicular cholestasis + mild lymphocytic inflammation and mild microvesicular steatosis	• Discontinuation of Pembrolizumab • Methylprednisolone 125 mg IV daily + MMF 1g/12h + Plasmapheresis daily up to 8 cycles	Death from acute liver failure
Doherty et al.	8 days after the first infusion of Pembrolizumab	• H&E staining: only a single small bile duct • Cytokeratin 7 immunohistochemistry staining: absence of bile ducts and typical autoimmune hepatitis-like features + very minimal and focal intermediate hepatobiliary phenotype	• Discontinuation of Pembrolizumab • Oral methylprednisolone 1mg/kg/día + MMF 1g/12h + UDCA	Death from progression of the underlying oncological disease
Doherty et al.	24 days after a single infusion of Pembrolizumab	Absence of bile ducts + severe cholestasis and duct injury with evidence of parenchymal loss and regeneration	• Discontinuation of Pembrolizumab • Methylprednisolone 2 mg/kg/day + MMF 500mg/12h + UDCA	Initial improvement in liver biochemistry. Later death from progression of the underlying oncological disease
Noblejas, Lazaro et al.	After the third infusion of Atezolizumab	Absence of bile ducts + intrahepatic cholestasis in all portal spaces and hepatic lobules	• Discontinuation of Atezolizumab • Methylprednisolone 250mg/day IV for three days (x2) + MMF 1g/12h + UDCA 15mg/kg/day	Progressive improvement of clinical and biochemical parameters (normal bilirubin levels after 6 months of treatment)

MMF, mycophenolate mofetil; UDCA, ursodeoxycholic acid; H&E, hematoxylin and eosin.

Signaling mediated by programmed death receptor-1 (PD-1) and its ligand (PD-L1) plays a key role in tumor evasion of the immune system. Although both PD-1 inhibitors and PD-L1 inhibitors share the goal of blocking this immunosuppressive pathway to restore the immune effector activity of T lymphocytes against tumor cells, they differ in their mechanism of action, leading to pathophysiological differences with relevant clinical implications.

Anti-PD-1 monoclonal antibodies bind directly to the PD-1 receptor, expressed on activated T lymphocytes, B lymphocytes, macrophages, regulatory T cells, and natural killer cells, blocking its interaction with both PD-L1 and PD-L2.

This dual inhibition enhances broader immune activation, although at the expense of a higher risk of immune tolerance disruption and immune-mediated adverse events (31).

In contrast, PD-L1 inhibitors such as Atezolizumab selectively bind to their ligand, which is expressed in tumor cells, stromal cells and antigen-presenting cells in the tumor microenvironment, allowing the interaction between PD-1 and PD-L2 to be preserved. Precisely, the degree of expression of PD-1 and PD-L2 in this microenvironment could influence the modulation of the immune response to these agents. Thus, this selectivity could contribute to the maintenance of immune tolerance and a tendency towards a potentially more favorable immune-mediated toxicity profile, without compromising antitumor therapeutic efficacy (32).

According to ESMO and NCCN guidelines (33, 34), atezolizumab is an adjuvant treatment for locally advanced non-small cell lung cancer. Although this therapy can cause unusual but serious adverse effects, as in this case, since it has been shown to induce complete and durable responses with a generally favorable safety profile, the therapeutic benefit clearly outweighs the associated risks. Therefore, in the event of a hypothetical tumor relapse, retreatment with Atezolizumab as an adjuvant could be considered, given the prolonged response and adequate clinical progression observed in this patient.

In conclusion, although immunotherapy has represented a significant advance in cancer treatment, its increasing use has revealed rare but potentially fatal adverse effects. In this context, we present the first documented case of Atezolizumab-induced vanishing bile duct syndrome, a finding of notable clinical relevance and with high impact on oncological pharmacovigilance, highlighting the need for a thorough evaluation of the hepatotoxic profile of this immunotherapeutic drug.

## Patient perspective

The patient’s experience, marked by intense anxiety stemming from uncertainty and diagnostic rarity, as well as prolonged clinical progression, highlights the importance of considering not only the clinical management of immune-mediated toxicity, but also its emotional impact throughout the therapeutic process, with a multidisciplinary approach and follow-up being essential.

## Data Availability

The original contributions presented in the study are included in the article/supplementary material. Further inquiries can be directed to the corresponding author.
